# Lower-extremity muscle strength is associated with prognosis in patients with advanced or recurrent lung cancer: a retrospective, observational study

**DOI:** 10.1186/s12885-025-13728-6

**Published:** 2025-02-18

**Authors:** Takuya Fukushima, Utae Katsushima, Naoya Ogushi, Kimitaka Hase, Jiro Nakano

**Affiliations:** 1 Faculty of Rehabilitation, Kansai Medical University, Hirakata, Osaka, Japan; 2 Department of Thoracic Oncology, Kansai Medical University, Osaka, Japan; 3 Department of Rehabilitation, Kansai Medical University Hospital, Osaka, Japan; 4 Department of Physical Medicine & Rehabilitation, Kansai Medical University, Osaka, Japan

**Keywords:** Lower-extremity muscle strength, Lung cancer, Prognosis, Sarcopenia

## Abstract

**Background:**

We aimed to examine associations between various sarcopenia indices—including skeletal muscle index (SMI), handgrip strength, lower-extremity muscle strength, a combined measure of handgrip and lower-extremity muscle strength, sarcopenia (defined as a combination of SMI and muscle strength), and the SARC–F questionnaire—and all-cause mortality in patients with advanced or recurrent lung cancer. Moreover, we aimed to identify factors influencing sarcopenia indices that demonstrate strong correlations with prognosis, aiming to inform the development of targeted interventional strategies.

**Methods:**

This retrospective observational study enrolled outpatients with lung cancer who underwent chemotherapy. Patients were evaluated for sarcopenia indices, including SMI, handgrip strength, five-repetition sit-to-stand test (5STS), and SARC–F. Physical activity was assessed using the International Physical Activity Questionnaire-Short Form (IPAQ–SF). The log-rank test and Cox proportional hazards model, adjusted for confounders, were used to examine the association between the sarcopenia index and prognosis. Harrell’s concordance index (C-index) was used to quantify the predictive power of the resultant model. To examine the significant factors associated with sarcopenia indices, which are associated with prognosis, multivariate logistic regression analysis was performed.

**Results:**

There was a significant association between low handgrip strength (hazard ratio [HR], 2.73; 95% confidence interval [CI], 1.20–6.25; *P* = 0.017), 5STS ≥ 12 s (low lower-extremity muscle strength) (HR, 2.32; 95% CI, 1.23–4.36; *P* < 0.01), the combination of low handgrip strength and 5STS ≥ 12 s (HR, 2.37; 95% CI, 1.23–4.57; *P* = 0.010), and sarcopenia (defined as a combination of SMI and muscle strength) (HR, 2.07; 95% CI, 1.02–4.21; *P* = 0.044) and survival, whereas there was no significant association between SMI (HR, 1.62; 95% CI, 0.74–3.53; *P* = 0.20) and SARC–F (HR, 2.07; 95% CI, 0.97–4.43; *P* = 0.061) and survival. The C-index for handgrip strength and 5STS was 0.625 (95% CI: 0.624–0.627) and 0.635 (95% CI: 0.634–0.636), respectively. Multivariate logistic analysis adjusted for age, sex, clinical stage, and treatment line showed that IPAQ–SF was an independent significant factor associated with 5STS ≥ 12 s (odds ratio [OR], 9.31; 95% CI, 2.93–29.58; *P* < 0.001), the combination of low handgrip strength and 5STS ≥ 12 s (OR, 6.45; 95% CI, 2.10–19.81; *P* = 0.001), and sarcopenia (OR, 4.90; 95% CI, 1.52–15.84; *P* = 0.008).

**Conclusions:**

Handgrip strength and lower-extremity muscle strength were stronger predictors of prognosis compared to the SMI. Furthermore, physical inactivity was significantly associated with lower-extremity muscle strength. From a clinical perspective, evaluating lower-extremity strength and physical activity is essential, and implementing exercise interventions, including strategies to enhance physical activity levels, should be considered.

## Background

Sarcopenia is a progressive and generalized skeletal muscle disorder characterized by accelerated loss of muscle mass and function [1]. Notably, it has been shown to shape treatment complications, dose-limiting toxicities, disease control rate, recurrence, and prognosis in several types of cancer [2, 3], in addition to decreasing function and activities of daily living (ADLs). Therefore, addressing sarcopenia is critical in the field of oncology.

Sarcopenia holds significant importance in patients with lung cancer, with approximately 50% affected by this condition [4], and approximately 20% exhibiting cachexia [5], which is defined as a multifactorial catabolic syndrome characterized by progressive loss of skeletal muscle mass (SMM) [6]. As observed in other cancers, meta-analyses have shown sarcopenia to be associated with prognosis in patients with lung cancer [4, 7–9]. While these reports define sarcopenia in terms of SMM, the SARC–F is recommended for screening, and muscle strength is included in the diagnosis of sarcopenia [1, 10]. More recently, as a factor, muscle strength has been incorporated into the official definition of the condition [11]. A previous study showed that low handgrip strength is associated with mortality in patients with lung cancer [12]. However, it remains unclear whether SMM or muscle strength measures are significantly associated with survival in patients with lung cancer. Additionally, reports examining muscle strength and prognosis have only examined handgrip strength and have not included lower-extremity muscle strength. Therefore, we hypothesized that identifying an index of sarcopenia with strong predictive value would inform effective treatment strategies.

We aimed to examine the relationship between various sarcopenia indices—such as SMM, handgrip strength, lower-extremity muscle strength, a combination of handgrip and lower-extremity muscle strength, sarcopenia (defined as the combination of skeletal muscle index [SMI] and muscle strength), and SARC–F—and prognosis in patients with advanced or recurrent lung cancer. Additionally, we aimed to identify factors influencing sarcopenia indices closely associated with prognosis, providing insights for potential interventional strategies.

## Methods

### Study design and population

This single-center retrospective observational study was conducted at Kansai Medical University (KMU). Patients were enrolled between April 2021 and December 2023. We included patients diagnosed with advanced or recurrent lung cancer, receiving chemotherapy in an outpatient setting, prescribed rehabilitation, scheduled for chemotherapy, and aged ≥ 20 years. Patients with communication difficulties, poor general status, or inability to undergo muscle function evaluation were excluded. Ethical approval was obtained from the Institutional Review Board of KMU (approval number: 2023264). An opt-out consent procedure was used owing to the retrospective nature of the study. All procedures were performed in accordance with the ethical standards of the committees responsible for human experimentation (institutional and national) and the Declaration of Helsinki of 1964 and its later versions.

### Measurements

We collected general and clinical information, including age, sex, body mass index (BMI), blood chemistry (C–reactive protein, hemoglobin, and albumin), Eastern Cooperative Oncology Group Performance Status [13], pathology, genetic mutation, clinical stage, bone and brain metastases, treatment, symptoms, physical activity, and nutritional status, from the medical records. The pathology was recorded as small or non-small-cell lung cancer. Clinical stages were categorized as II–IIIA or IIIB–IV, and recurrence was based on the eighth tumor–node–metastasis classification of the Union for International Cancer Control. Information regarding the treatment lines (first, second, third, or higher) was collected. The presence or absence of numbness, pain, and dyspnea was recorded as symptoms. At the start of rehabilitation, the Japanese version of the International Physical Activity Questionnaire–Short Form (IPAQ–SF) was used to assess physical activity [14]. The IPAQ–SF is a questionnaire-based assessment that measures weekly energy expenditure (min/week) and provides individuals with a continuous weekly physical activity level. Physical activity was defined as the total amount of physical activity (walking, moderate-intensity, and vigorous–intensity) based on a previous study [15]. The Mini Nutritional Assessment Short Form (MNA–SF) was used to measure the nutritional status [16]. This tool consists of six domains: food intake, weight loss, gait, mental stress, acute illness, neurological and psychological problems, and BMI. Each item was scored on a scale of 0–2 or 3, and a total score of < 8 points was defined as malnutrition [17].

#### SMM

SMM was measured using a multifrequency segmental body composition analyzer (TANITA MC–780A–N; TANITA Co., Ltd., Tokyo, Japan). For the SMI, the appendicular SMM was summed and divided by the square of the height. Low SMI was defined as < 7.0 kg/m^2^ for men and < 5.7 kg/m^2^ for women [10].

#### Handgrip strength

Handgrip strength was measured using a standard adjustable handle dynamometer (TKK 5101; Takei Scientific Instruments Co., Ltd., Niigata, Japan). Measurements were performed with the patient standing, arms held at zero abduction, and flush against the body. The maximum value from a single attempt was recorded in kilograms (kg). Low handgrip strength was defined as < 28.0 kg for men and < 18.0 kg for women [10].

#### Lower-extremity muscle strength

The five-repetition sit-to-stand test (5STS) was used as an indicator of lower-extremity muscle strength [18]. The time taken to stand up from a chair (45 cm high) five times as quickly as possible without using the arms was measured with the patient’s arms crossed over the chest. Low lower-extremity muscle strength was defined as ≥ 12 s [10].

#### Sarcopenia

Sarcopenia was defined according to the Asian Working Group for Sarcopenia (AWGS) 2019 criteria [10], incorporating low SMI, low handgrip strength, and low lower-extremity muscle strength. The specific cutoff values for SMI, handgrip strength, and lower-extremity muscle strength are outlined above.

#### SARC–F

The SARC–F includes five components: strength, assistance in walking, rising from a chair, climbing stairs, and falling. SARC–F items were reported on a 3-point scale (0, 1, 2), and all items were summed to calculate the SARC–F score (range, 0–10). Four or more items were considered positive during the screening [19, 20].

### Statistical analysis

Data were summarized as medians (interquartile ranges) or as counts and percentages. Survival days were calculated from the initiation of outpatient rehabilitation to either the date of death (all-cause mortality) or the last follow-up visit, whichever occurred first. Kaplan–Meier curves and log-rank tests were employed to evaluate the impact of sarcopenia-related indices—SMI, handgrip strength, 5STS, a combination of handgrip strength and 5STS, sarcopenia (defined by AWGS 2019 as a combination of SMI and muscle strength), and SARC–F—on survival outcomes.

A Cox proportional hazards model, adjusted for clinically relevant factors identified in previous research, including age, sex, pathology, treatment line, and clinical stage [21–23], was utilized to further assess the influence of sarcopenia indices on survival. Model discriminative power was evaluated using Harrell’s concordance index (C-index). Receiver operating characteristic analysis was conducted to determine the cutoff value of IPAQ–SF for 5STS ≥ 12 s [24].

Multivariate logistic regression analysis was performed to identify factors significantly associated with sarcopenia indices, which are associated with prognosis, incorporating variables such as BMI, IPAQ–SF, MNA–SF, numbness, pain, and dyspnea, with adjustments for age, sex, treatment line, and clinical stage. Statistical significance was defined as P < 0.05. Analyses were conducted using R software (version 4.4.0; R Foundation for Statistical Computing, Vienna, Austria).

## Results

### Patient characteristics

**Fig. 1:**
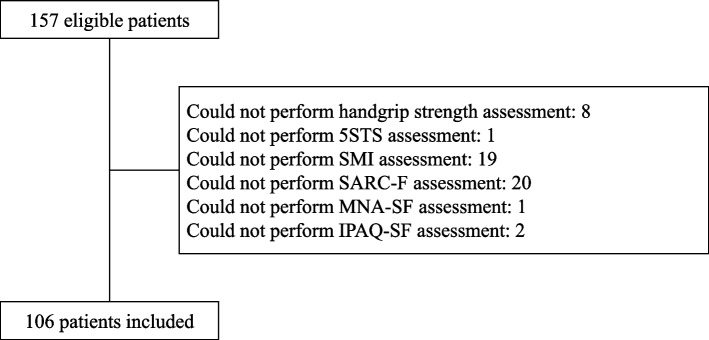
Flow diagram

Of the 157 patients with advanced or recurrent lung cancer, 51 were excluded from the analysis, leaving 106 patients in the study (Fig. 1). Table 1 summarizes the general and clinical characteristics of these patients. The median age was 75.0 years (interquartile range: 71.0–79.0 years), and 29 patients (27.4%) were female. Among the cohort, 83 patients (78.3%) had non-small-cell lung cancer, and 23 (21.7%) had a genetic mutation.

**Table 1:** Baseline and clinical characteristics data

Characteristics	*n* = 106
Age, median [IQR], years	75.0 [71.0 to 79.0]
Sex, no. (%), Male / Female	77(72.6) / 29(27.4)
BMI, median [IQR], kg/m^2^	21.0 [18.8 to 23.7]
CRP, median [IQR], mg/dl	0.60 [0.17 to 1.33]
Hemoglobin, median [IQR], g/dl	11.2 [9.5 to 12.2]
Albumin, median [IQR], g/dl	3.8 [3.3 to 4.0]
ECOG–PS, no. (%)	
0	2(1.9)
1	89(84.0)
2	14(13.2)
3	1 (0.9)
Pathology, no. (%)	
Small	23(21.7)
Non-small	83(78.3)
Genetic mutation, no. (%)	
None	77(72.6)
EGFR	15(14.2)
KRAS	5(4.7)
ROS1	2(1.9)
SMARCA4 deficiency	1(0.9)
Unknown	6(5.7)
PD-L1 tumor proportion score, no. (%)	
< 1%	18(17.0)
1–49%	18(17.0)
≥ 50%	11(10.4)
Unknown	59(55.6)
Clinical stage, no. (%)	
II-IIIA	15(14.2)
IIIB-IV and recurrence	91(85.8)
Bone metastases, no. (%)	24(22.6)
Brain metastases, no. (%)	13(12.3)
Treatment line, no. (%)	
First	75(70.8)
Second	19(17.9)
Third	12(11.3)
Treatment details, no. (%)	
Chemotherapy + ICI	39(36.8)
Chemotherapy	29(27.4)
ICI	18(17.0)
Molecular targeted therapy	6(5.7)
Chemotherapy + Molecular targeted therapy	5(4.7)
Others	9(8.4)
Numbness, no. (%)	29 (27.4)
Pain, no. (%)	25 (23.6)
Dyspnea, no. (%)	63 (59.4)
IPAQ–SF, median [IQR], METs–min/week	280.5 [0.0 to 717.8]
MNA–SF, median [IQR]	11.0 [9.0 to 13.0]
SMI, median [IQR], kg/m^2^	6.29 [5.75 to 7.04]
Low SMI, no. (%)	61 (57.5)
Handgrip strength, median [IQR], kg	23.6 [19.1 to 27.8]
Low handgrip strength, no. (%)	67 (63.2)
5STS, median [IQR], s	9.35 [7.69 to 11.78]
5STS ≥ 12 s (low lower extremity muscle strength)	29 (27.4)
SARC–F, median [IQR]	1 [0 to 3]
SARC–F ≥ 4	17 (16.0)

*5STS* Five–repetition sit–to–stand test, *BMI* Body mass index, *CRP* C–reactive protein, *ECOG–PS* Eastern Cooperative Oncology Group Performance Status, *ICI* Immune checkpoint inhibitors, *IPAQ–SF* International Physical Activity Questionnaire–Short Form, *IQR* Interquartile range, *MNA–SF* Mini Nutritional Assessment Short Form, *SMI* Skeletal muscle index

The majority, 91 patients (85.8%), were diagnosed with stage IIIB–IV disease or recurrence; 24 patients (22.6%) had bone metastases, and 13 (12.3%) had brain metastases. First-line treatment had been administered to 75 patients (70.8%), while 39 patients (36.8%) had undergone chemotherapy combined with immune checkpoint inhibitors.

The median IPAQ–SF score was 280.5 METs–min/week (interquartile range: 0.0–717.8 METs–min/week), and the median MNA–SF score was 11 (interquartile range: 9–13). Sarcopenia-related findings included 61 patients (57.5%) with low SMI, 67 (63.2%) with low handgrip strength, 29 (27.4%) with low lower-extremity muscle strength (5STS ≥ 12 s), and 17 (16.0%) with SARC–F ≥ 4.

### Association between the sarcopenia index and prognosis

**Fig. 2:**
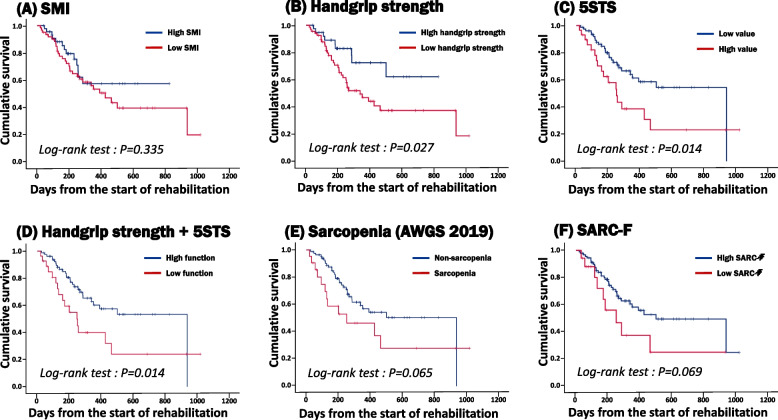
Association between sarcopenia index and prognosis

Kaplan–Meier curves were generated to evaluate the impact of sarcopenia-related indices, including SMI, handgrip strength, 5STS, combination of handgrip strength and 5STS, sarcopenia (combination of SMI and muscle strength), and SARC–F, on survival. Log-rank tests revealed significant differences in survival for handgrip strength (*P* = 0.027), 5STS (*P* = 0.014), and the combination of handgrip strength and 5STS (*P* = 0.014). No significant survival differences were observed for SMI (*P* = 0.335), sarcopenia (combination of SMI and muscle strength) (*P* = 0.065), or SARC–F (*P* = 0.069) (Fig. 2).

In the Cox proportional hazards model adjusted for potential confounding variables, including age, sex, pathology, treatment line, and clinical stage, the following indices were significantly associated with survival: low handgrip strength (hazard ratio [HR], 2.73; 95% confidence interval [CI], 1.20–6.25; *P* = 0.017), 5STS ≥ 12 s (low lower-extremity muscle strength) (HR, 2.32; 95% CI, 1.23–4.36; *P* = 0.009), the combination of low handgrip strength and 5STS ≥ 12 s (HR, 2.37; 95% CI, 1.23–4.57; *P* = 0.010), and sarcopenia defined by AWGS 2019 criteria (HR, 2.07; 95% CI, 1.02–4.21; *P* = 0.044). Conversely, SMI (HR, 1.62; 95% CI, 0.74–3.53; *P* = 0.230) and SARC–F (HR, 2.07; 95% CI, 0.97–4.43; *P* = 0.061) were not significantly associated with survival (Table 2).

**Table 2:** Association between sarcopenia index and prognosis

Parameters (reference)	Model: I			Model: II			Model: III			Model: IV			Model: V			Model: VI		
	HR	95%CI	*P*-value	HR	95%CI	*P*-value	HR	95%CI	*P*-value	HR	95%CI	*P*-value	HR	95%CI	*P*-value	HR	95%CI	*P*-value
SMI, below cut-off^a^ (above cut-off)	1.62	0.74–3.53	0.230															
Handgrip strength, below cut-off^b^ (above cut-off)				2.73	1.20–6.25	0.017												
5STS, ≥ 12 s (< 12 s)							2.32	1.23–4.36	0.009									
Handgrip strength + 5STS, low function^c^ (high function)										2.37	1.23–4.57	0.010						
Sarcopenia (AWGS 2019), yes (no)													2.07	1.02–4.21	0.044			
SARC-F, ≥ 4 (< 4)																2.07	0.97–4.43	0.061
Age	0.99	0.95–1.03	0.577	0.98	0.94–1.02	0.380	0.98	0.95–1.02	0.406	0.98	0.95–1.02	0.333	0.99	0.95–1.02	0.479	0.99	0.95–1.03	0.631
Sex, male (female)	1.04	0.47–2.27	0.927	1.04	0.50–2.16	0.910	1.30	0.64–2.66	0.465	1.24	0.61–2.53	0.558	1.22	0.60–2.51	0.585	1.44	0.69–3.03	0.333
Pathology, small (non-small)	1.02	0.48–2.17	0.949	0.96	0.46–2.03	0.919	0.91	0.43–1.92	0.801	0.89	0.42–1.88	0.751	0.91	0.43–1.94	0.804	1.04	0.49–2.21	0.910
Stage, IIIB–IV and recurrence (others)	1.27	0.44–3.67	0.657	0.93	0.31–2.80	0.904	1.33	0.46–3.81	0.600	1.29	0.45–3.71	0.639	1.35	0.47–3.88	0.584	1.30	0.45–3.76	0.624
Treatment line, third (others)	1.99	0.77–5.14	0.154	2.06	0.84–5.09	0.116	1.76	0.73–4.26	0.211	1.70	0.70–4.11	0.238	1.87	0.76–4.63	0.176	1.59	0.65–3.87	0.309

*5STS* Five–repetition sit–to–stand test, *AWGS* Asian Working Group for Sarcopenia, *CI* Confidence Interval, *HR* Hazard ratio, *SMI* Skeletal muscle index

^a^< 7.0 kg/m^2^ for men and < 5.7 kg/m^2^ for women

^b^< 28.0 kg for men and < 18.0 kg for women

^c^Handgrip strength < 28.0 kg for men and < 18.0 kg for women, 5STS ≥ 12 s

### Predictive performance of sarcopenia index for survival in patients with lung cancer

The C-index values for the sarcopenia-related indices were as follows: handgrip strength, 0.625 (95% CI: 0.624–0.627); 5STS, 0.635 (95% CI: 0.634–0.636); combination of handgrip strength and 5STS, 0.640 (95% CI: 0.639–0.641); SMI, 0.565 (95% CI: 0.564–0.566); sarcopenia (combination of SMI and muscle strength), 0.622 (95% CI: 0.621–0.623); and SARC–F, 0.588 (95% CI: 0.587–0.589).

### Factors influencing sarcopenia index associated with prognosis

Multivariate logistic analysis adjusted for age, sex, clinical stage, and treatment line showed that IPAQ–SF was an independent significant factor associated with 5STS ≥ 12 s (low lower-extremity muscle strength) (odds ratio [OR], 9.31; 95% CI, 2.93–29.58; *P* < 0.001), the combination of low handgrip strength and 5STS ≥ 12 s (OR, 6.45; 95% CI, 2.10–19.81; *P* = 0.001), and sarcopenia (defined as a combination of SMI and muscle strength) (OR, 4.90; 95% CI, 1.52–15.84; *P* = 0.008) (Table 3). No factors were associated with low handgrip strength.

**Table 3:** Factors influencing sarcopenia index associated with prognosis

Parameters (reference)	Low handgrip strength			5STS ≥ 12 s (low lower extremity muscle strength)			Low handgrip strength + 5STS ≥ 12 s (low lower extremity muscle strength)			Sarcopenia as defined by AWGS2019		
	OR	95%CI	*P*-value	OR	95%CI	P-value	OR	95%CI	*P*-value	OR	95%CI	*P*-value
BMI	0.94	0.82–1.08	0.391	0.99	0.83–1.18	0.919	1.01	0.84–1.21	0.918	0.99	0.82–1.20	0.907
IPAQ-SF, < 16.5 (≥ 16.5)	2.32	0.79–6.83	0.124	9.31	2.93–29.58	< 0.001	6.45	2.10–19.81	0.001	4.90	1.52–15.84	0.008
MNA-SF, < 8 (≥ 8)	2.10	0.46–9.56	0.337	3.40	0.80–14.39	0.097	4.00	0.95–16.87	0.059	3.94	0.90–17.34	0.070
Numbness, yes (no)	0.88	0.31–2.53	0.814	2.35	0.70–7.89	0.168	1.87	0.56–6.26	0.313	1.59	0.45–5.59	0.469
Pain, yes (no)	1.55	0.49–4.89	0.456	0.98	0.26–3.74	0.975	1.20	0.32–4.53	0.787	1.29	0.33–5.04	0.711
Dyspnea, yes (no)	0.65	0.24–1.77	0.400	0.67	0.22–2.07	0.483	0.83	0.27–2.57	0.746	0.79	0.24–2.60	0.696

Multivariate logistic analysis was adjusted for age, sex stage, and treatment line

*5STS* Five–repetition sit–to–stand test, *AWGS* Asian Working Group for Sarcopenia, *BMI* Body mass index, *CI* Confidence Interval, *IPAQ–SF* International Physical Activity Questionnaire–Short Form, *MNA–SF* Mini Nutritional Assessment Short Form, *OR* Odds ratio

## Discussion

The clinical relevance of sarcopenia extends beyond impacting ADLs; it also shapes the prognosis of patients with lung cancer. However, it remains unclear whether SMM or muscle strength measures are stronger predictors of survival. Solving this problem is crucial for developing effective treatment strategies for patients with lung cancer. Therefore, we investigated and compared the relationship between sarcopenia indices, including SMI, handgrip strength, lower-extremity muscle strength, SARC–F, and prognosis. To our knowledge, this is the first study to investigate various sarcopenia indices with high prognostic accuracy in patients with advanced or recurrent lung cancer.

In this study, the SARC–F score, a screening tool for sarcopenia, was not associated with prognosis. Mori et al. reported that the SARC–F is a predictor of prognosis in patients with cancer, but no significant association was found with lung cancer [25]. This warrants more comprehensive assessments of muscle function, in addition to screening, to predict the prognosis of lung cancer.

A previous meta-analysis showed that sarcopenia, as defined by the SMM, affects the prognosis of patients with lung cancer [4, 7, 8]. Contrary to our assumptions, no significant association was observed between SMI and prognosis in this study. This result may be attributable to patient-specific characteristics. Yang et al. [4], Deng et al. [7], and Buentzel et al. [9] included early-stage cancers in their analyses, and the prevalence of low SMM was not extremely high. It is well known that the prevalence of sarcopenia increases with the progression of cancer [2]. Our study only included patients with advanced or recurrent cancers, and the prevalence of low SMI was approximately 60%. Thus, many patients had low SMI; therefore, SMM alone may not critically impact prognosis. A previous study demonstrated that incorporating muscle strength and physical performance into SMM measurements enhanced prognostic accuracy in patients with lung cancer [26]. However, in this study, adding muscle strength or physical performance to low SMI did not yield a significant improvement in prognostic ability, as low SMI alone was not significant in the log-rank test. Nevertheless, the Cox proportional hazards model highlighted the prognostic significance of sarcopenia (a combination of SMI and muscle strength).

Interestingly, muscle strength, rather than SMI, was significantly associated with prognosis in this study. Previous studies have shown that muscle strength is a stronger predictor of prognosis than SMM in gastrointestinal and hepatobiliary cancers [27] and incurable cancers [28], which supports our results. Recently, muscle-specific strength was included in the conceptual definition of sarcopenia by the Global Leadership Initiative in Sarcopenia [11]. This indicates the importance of increasing muscle strength with limited muscle mass. Both handgrip strength and 5STS (lower-extremity muscle strength) were significantly associated with prognosis after adjusting for covariates. A previous study demonstrated that low handgrip strength is associated with increased mortality in patients with lung cancer [12]. Regarding the prognostic value of upper- and lower-extremity muscle strength, Yasunobe et al. reported that lower-extremity muscle strength is a stronger predictor of prognosis compared to handgrip strength [29]; however, no prior studies have evaluated this in patients with lung cancer. This study's finding that lower-extremity muscle strength is equally predictive of prognosis as upper-extremity muscle strength represents a novel contribution to the field. Notably, lower-extremity muscle strength is linked to the continuity of outpatient treatment, suggesting its relevance to patient outcomes.

While the prognostic abilities of upper- and lower-extremity muscle strength were comparable, the reasons underlying these similarities remain unclear. Furthermore, the combined assessment of both upper- and lower-extremity muscle strength did not show an additive benefit over their individual prognostic abilities. This intriguing observation warrants further investigation to elucidate the mechanisms behind these findings.

For 5STS ≥ 12 s (low lower-extremity muscle strength), the combination of low handgrip strength and 5STS ≥ 12 s, and sarcopenia (defined as a combination of SMI and muscle strength), physical inactivity was an independent significant factor in this study. These sarcopenia indices are characterized by the inclusion of lower extremity muscle strength. Physical activity is reportedly associated with lower-extremity muscle strength [30], which has also been shown in hematological malignancies [31], supporting our findings. However, to our knowledge, no reports have examined physical activity and lower-extremity muscle strength-related sarcopenia indices in patients with advanced lung cancer. Although the failure to find treatment implications for handgrip strength remains a challenge, the results of this study will be of interest to clinical applications. Lower-extremity muscle strength should be assessed, and physical activity should be quantified in patients with lung cancer. If lower-extremity muscle weakness is present, functional improvement should be achieved through exercise therapy, including increased physical activity. Future studies are needed to determine whether improvement in lower-extremity muscle strength contributes to prognosis.

This study had several limitations. First, the possibility of selection bias cannot be excluded, as the study only included cases where rehabilitation was prescribed, and sarcopenia could be assessed. Second, being a single-center, retrospective observational study, the design may impact the reproducibility, robustness, and generalizability of the findings. Third, as this was an exploratory observational study, a formal sample size calculation was not performed. The relatively small sample size further limits the study's conclusions. Fourth, the subgroup of 17 patients with SARC–F ≥ 4 may lack sufficient statistical power, potentially affecting the reliability of conclusions. Larger multicenter studies are needed to validate and generalize these findings. Fifth, the study predominantly included male participants, leaving uncertainty regarding the applicability of the results to women. Sixth, only patients who underwent sarcopenia index assessments were analyzed, which may introduce additional selection bias. Finally, oncological heterogeneity—including variations in pathology, disease characteristics, and treatments—could influence the results. While efforts were made to statistically adjust for these factors, they remain potential confounders.

## Conclusions

Our findings showed that not only handgrip strength but also lower-limb muscle strength was a stronger predictor of prognosis than SMM. Furthermore, physical inactivity was significantly associated with lower-extremity muscle strength.

Clinically, it is essential to assess lower-extremity strength and physical activity, as these factors are critical for patient prognosis. Exercise interventions, including strategies to enhance physical activity levels, should be considered as part of a comprehensive approach to patient care.

## Data Availability

The data that support the findings of this study are available from the corresponding author upon reasonable request.

## Abbreviations

5STS: Five-repetition sit-to-stand test
ADL: Activities of daily living
BMI: Body mass index
CI: Confidence interval
ECOG–PS: Eastern Cooperative Oncology Group Performance Status
HR: Hazard ratio
IPAQ–SF: International Physical Activity Questionnaire–Short Form
KMU: Kansai Medical University
MNA–SF: Mini Nutritional Assessment Short Form
SMI: Skeletal muscle index
SMM: Skeletal muscle mass
